# Tuning the Metal–Insulator Transition Properties of VO_2_ Thin Films with the Synergetic Combination of Oxygen Vacancies, Strain Engineering, and Tungsten Doping

**DOI:** 10.3390/nano12091470

**Published:** 2022-04-26

**Authors:** Mohamed A. Basyooni, Mawaheb Al-Dossari, Shrouk E. Zaki, Yasin Ramazan Eker, Mucahit Yilmaz, Mohamed Shaban

**Affiliations:** 1 Department of Nanotechnology and Advanced Materials, Graduate School of Applied and Natural Science, Selçuk University, Konya 42030, Turkey; m.a.basyooni@gmail.com (M.A.B.); shrouk.e.zaki@gmail.com (S.E.Z.); 2 Department of Nanoscience and Nanoengineering, Institute of Science and Technology, University of Necmettin Erbakan, Konya 42060, Turkey; mucahityilmaz@erbakan.edu.tr; 3 Science and Technology Research and Application Center (BITAM), University of Necmettin Erbakan, Konya 42060, Turkey; 4 Research Center for Advanced Materials Science (RCAMS), King Khalid University, Abha 61413, Saudi Arabia; mdosri@kku.edu.sa; 5 Department of Physics, Dhahran Aljanoub, King Khalid University, Abha 61421, Saudi Arabia; 6 Department of Metallurgy and Material Engineering, Faculty of Engineering and Architecture, Necmettin Erbakan University, Konya 42060, Turkey; 7 Department of Physics, Faculty of Science, Islamic University of Madinah, AlMadinah Almonawara 42351, Saudi Arabia; 8 Nanophotonics and Applications Laboratory, Physics Department, Faculty of Science, Beni-Suef University, Beni-Suef 62514, Egypt

**Keywords:** thin films, vanadium oxide, thermochromic, phase transition device, metal–insulator transition

## Abstract

Vanadium oxide (VO_2_) is considered a Peierls–Mott insulator with a metal–insulator transition (MIT) at T_c_ = 68° C. The tuning of MIT parameters is a crucial point to use VO_2_ within thermoelectric, electrochromic, or thermochromic applications. In this study, the effect of oxygen deficiencies, strain engineering, and metal tungsten doping are combined to tune the MIT with a low phase transition of 20 °C in the air without capsulation. Narrow hysteresis phase transition devices based on multilayer VO_2_, WO_3_, Mo_0.2_W_0.8_O_3,_ and/or MoO_3_ oxide thin films deposited through a high vacuum sputtering are investigated. The deposited films are structurally, chemically, electrically, and optically characterized. Different conductivity behaviour was observed, with the highest value towards VO_1.75_/WO_2.94_ and the lowest VO_1.75_ on FTO glass. VO_1.75_/WO_2.94_ showed a narrow hysteresis curve with a single-phase transition. Thanks to the role of oxygen vacancies, the MIT temperature decreased to 35 °C, while the lowest value (T_c_ = 20 °C) was reached with Mo_0.2_W_0.8_O_3_/VO_2_/MoO_3_ structure. In this former sample, Mo_0.2_W_0.8_O_3_ was used for the first time as an anti-reflective and anti-oxidative layer. The results showed that the MoO_3_ bottom layer is more suitable than WO_3_ to enhance the electrical properties of VO_2_ thin films. This work is applied to fast phase transition devices.

## 1. Introduction

VO_2_ is one of the simplest correlated oxide materials, with a metal–insulator transition (MIT) phase transition at 68 °C [1]. The semiconductor phase was observed at low temperatures with a monoclinic crystal structure. In contrast, at high temperatures, it is a tetragonal rutile structure [2]. VO_2_ is the most intensively investigated solid-state thermochromic, metamaterials, and switching terahertz device [3,4,5]. It is still not ready for industrial or commercial application due to several problems. For instance, the transition temperature at 68 °C is too high for applications in daily life (such as intelligent window applications). This issue can be overcome since the conductivity of VO_2_ can be changed with different doping elements, which can control the phase transition (T_c_) value. For example, W^6+^ and Mo^6+^ increase the electron concentrations by tending to the metallic state, decreasing the T_c_ [6,7]. Despite the considerable difference in the elementary particle amount between elements W74184 & M4296o), they can contribute similarly to the VO_2_ structure. Based on the Hume-Rothery rules, VO_2_, MoO_3_, and WO_3_ can crystallize in the same or compatible structure. Simultaneously, +6 oxidization state, the ionic radii of these nuclei are close to Mo^6+^: 62 pm, W^6+^: 60 pm, and V^4+^: 58 pm. On the other hand, elements with lower valence, such as Cr^3+^ or Al^3+^, have also been used as doping elements [8]. The luminous transmittance of VO_2_ at 50% (or less) is too low for daily use, which should be increased for real applications. Moreover, the solar energy modulation between the VO_2_′s two states is modest, which should be considered. Research efforts are targeted at overcoming the above issues. For instance, T_c_ can be modulated to a suitable temperature via doping, which depends on many factors such as the charge and size of the dopant ion and the change of electron carrier density. A multilayer structure is adopted to improve the luminous transmittance of VO_2_ coating. However, enhancing these factors together requires to combine effects such as oxygen vacancies and strain engineering, as reported below.

Oxygen vacancies on transition metal oxide surfaces (or in bulk) can alter the host system’s electronic structure and chemical properties, even influencing VO_2_ electric properties. As a result, the properties of the vacancies would be primarily determined by the host system. The defect reaction defines the formation of an oxygen vacancy $O_{O}^{X}\to \frac{1}{2}O_{2}\left(g\right)\;+\;V_{O}^{••}\;+\;2e^{\prime }$. When the host crystal contains a cation with several stable oxidation states, the reaction will be of the type $O_{O}^{X}\;+\;M_{M}^{X}\to \frac{1}{2}O_{2}\left(g\right)\;+\;V_{O}^{••}\;+\;2M_{M}{}^{\prime }$. To maintain charge neutrality, an oxygen vacancy can be formed by delocalized or localized compensation by donating electrons to the conduction band or reducing cations in the host material, respectively [9]. There are still difficulties in the experimental determination of the vacancy-induced lattice relaxations and information about the vacancy formation energy (hence defect stability) in VO_2_ and V_2_O_5_ [10]. The electron doping induced by oxygen vacancies suppressed the MIT’s lower temperature in oxygen-deficient VO_2_ [11]. It can decrease the transition temperature to room temperature or even lower [12]. Zhang et al. reported a low-temperature oxidation annealing process (LTP) to prolong the oxygenation time of the oxygen-deficient VO_2_, to understand the role of oxygen vacancy in the structure and MIT of VO_2_ [13]. Since VO_2−*x*_ is more strain-sensitive than pure VO_2_, combining the effects of oxygen vacancy and compressive strain could effectively tune phase transition behaviour and lower phase transition temperatures [14]. For 2D Van der Waals (VDWs) structures, VO_2_ plays a prominent role in compressive strain, resulting in a shift in Raman modes of MoS_2_, especially in the photoluminescence of trion peak to lower energy [15].

Moreover, the role of grain boundaries on the MIT of VO_2_ is also interesting. By combining plasmon resonance nano spectroscopy with density functional calculations, Appavoo et al. studied the role of defects in the MIT on lithographically defined VO_2_ nanoparticles. They showed that the most likely point defect nucleating the phase transition in MIT is an oxygen vacancy present at grain boundaries created by strain [16].

In this work, the preparation and characterization of multilayer WO_3_, Mo_0.2_W_0.8_O_3,_ and MoO_3_ in contact with monoclinic VO_2_ (M–VO_2_) is reported. Optical, electrical, structural, morphological, and topographical characterizations are performed. This study is concerned with tuning the electrical properties and the temperature phase transition of monoclinic VO_2_ thin film on glass substrates for phase-change devices and applications. The effect of oxygen vacancy, strain engineering, and metal tungsten doping were simultaneously combined to tune the Metal–Insulator Transition (MIT) for the first time based on multilayer thin films. Many combinations based on VO_2_, WO_3_, Mo_1−*x*_W*_x_*O_3_, and/or MoO_3_ thin films are covered here to combine the effects of oxygen vacancy, strain effects, and doping effects. Due to the high optical transmission of Mo_1−*x*_W*_x_*O_3,_ these layers are inserted at the top of the structure to work as an anti-reflection layer and anti-oxidation, as reported in our recent study [17]. To obtain information about the oxidation state of the prepared monoclinic VO_2_ layer, several characterizations such as X-ray photoelectron spectroscopy (XPS) are reported.

## 2. Materials and Methods

### 2.1. Preparation of Thin Films

**Figure 1:**
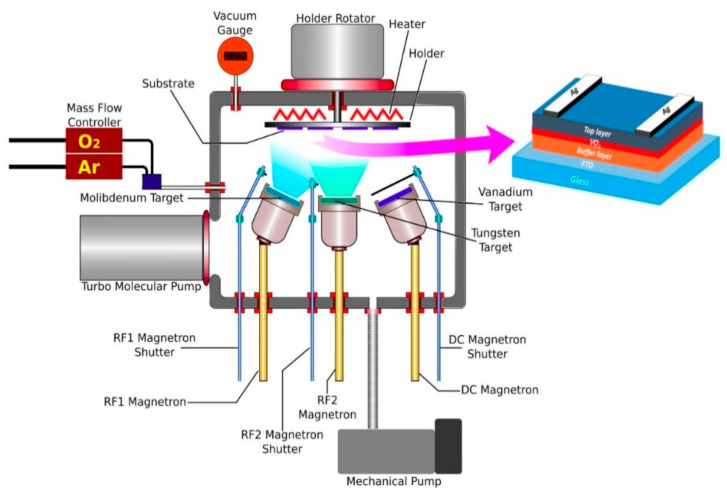
A schematic diagram for the used RF and DC sputtering system.

The samples were produced in a VAKSİS-MİDAS-3M1T magnetron sputter system (VAKSIS, Ankara, Turkey). Figure 1 shows the schematic diagram of the multilayer structure of the device. There are two 13.56 MHz power supplies and one DC power supply in the system with three magnetrons. Multilayer thin films were fabricated from 3 inch and 99.9% purity vanadium, molybdenum, and tungsten metallic targets by reactive sputtering. Argon and oxygen gases connected to the magnetron sputter system had a purity of 99.98%. Before starting the sputtering process, the FTO substrates were cleaned with acetone and isopropyl alcohol by keeping them in an ultrasonic cleaner for 15 min. They were then rinsed with deionized water and dried with high-purity nitrogen gas. After the substrates placed in the magnetron sputter holder were placed into the system, the system started to vacuum and was reduced to a 9.3 × 10^−7^ mbar pressure level. After reaching the desired pressure level, the sample holder was heated to 400 °C. It was kept at this temperature for 30 min for the temperature to stabilize on the substrate. Abbreviations of sample names and all deposition parameters are summarized in Table 1. The system pressure was increased to 6.8 × 10^−3^ mbar for the reactive sputter process for all samples. Time-dependent thickness optimization was achieved by using samples that had been produced in different thicknesses and whose thickness measurements had been made before. This optimization was used in sample fabrications as shown in Table 1. By controlling the film deposition times, the thicknesses of V–O, W–O, Mo–O, and Mo–W–O films were kept constant for all samples. The Mo_0.2_W_0.8_O_3_ thin film is a composite film of Mo and W, in the presence of a 12.1 sccm oxygen environment. Whereas, the Ar flow is 50 sccm. The Mo and W powers are 27 and 110 W, respectively. The deposition time was 16.7 min during the whole process for both Mo and W targets. The idea here is to introduce a small amount of Mo atoms into the WO_3_ film to form a high-quality anti-refractive anti-oxidative layer. In addition, this layer provides high transmission and good electrical performance, as predicted from our previous work [15,17]. The optimization of the content of Mo into WO_3_ has been covered before [17,18].

### 2.2. Characterization Techniques

The optical transmission (T) result was measured using UV-VIS-NIR Spectrophotometer (UV-3600i Plus, Shimadzu, Tokyo, Japan). The topography of the films was studied using Atomic Force Microscopy Park XE7 system (Park, Santa Clara, CA, USA) with a scanning area of 1 × 1 μm^2^, and a tip scan speed of 0.5 Hz, through noncontact mode. The crystal structures were analysed using X-ray diffraction GNR ADP PRO 2000 (GNR Srl, Novara, Italy) with a radiation source of CuK_α_ (λ = 1.5405 Å) step of 0.01. These data were refined via the Rietveld method applied using Fullprof suite software (Version 5 January 2021) to determine the exact metal-oxygen concentrations of each layer and different phases. The surface morphology was recorded using field-emission scanning electron microscopy (FESEM, GeminiSEM 500, ZEISS, Cambridge, UK). The energy dispersive X-ray (EDX) analysis of the samples was carried out to characterize the quantitative analyses of the samples using an Oxford, Xmax 50 SEM (Oxford Instruments, Oxford, UK) attached with an Oxford EDS detector. Raman vibrational modes were obtained using Renishaw inVia confocal Raman microscope (Renishaw, New Mills, UK) using 532 nm laser beam with 10 mW laser power. X-ray photoelectron spectroscopy (XPS) measurements were carried out on the Thermo Scientific K-alpha XPS system (Thermo Scientific™, Waltham, MA, USA) using an AlK_α_ source and a spot size of 400 µm. Fourier Transform Infrared Spectrometer (FTIR) through a 200–4000 nm range were collected with Thermo Scientific-Nicolet iS20 (Waltham, MA, USA). The electrical I–V phase transition measurements were taken using a 2450 Keithley Source Meter (Tektronix, Beaverton, OR, USA) and a four-probe system in which a heating stage is connected. The thicknesses of the samples were measured using the thin film analyzer system F20-UV (FILMETRICS, San Diego, MA, USA) with a ±1 nm error. The values are as follows: S1 (VO_2_) is 50.651 nm, S2 (VO_2_/MoO_3_) is 150.726 nm, S3 (VO_2_/WO_3_) is 148.908 nm, S4 (WO_3_/VO_2_/MoO_3_) is 248.983 nm, S5 (Mo_0.2_W_0.8_Os_3_/VO_2_/MoO_3_) is 269.302 nm, and S6 (Mo_0.2_W_0.8_O_3_/VO_2_ + W/MoO_3_) is 276.672 nm.

## 3. Results and Discussion

### 3.1. Characterization of FTO Supported Oxide Thin Film

**Figure 2:**
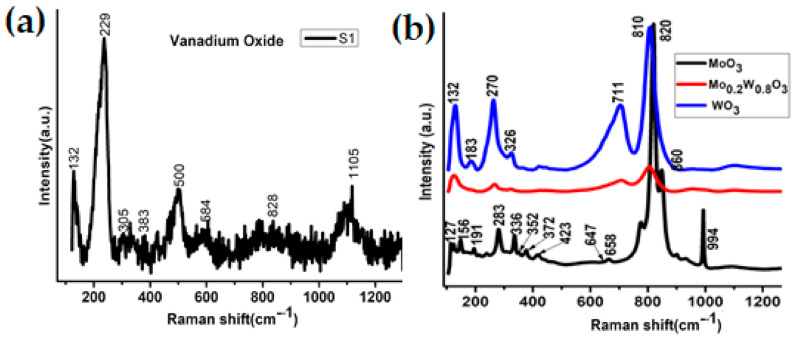
Raman spectra of the one-layer-based phase transition devices. (**a**) Characteristic Raman peaks of vanadium oxide (S1). (**b**) Characteristic Raman peaks of tungsten trioxide, molybdenum trioxide, and molybdenotungsten trioxide thin films.

The samples studied in this work are based on the successive deposition of metal oxide thin film. Therefore, the preparation conditions regarding each desired layer on FTO glass have been optimized. Typically used, the Raman spectra show the successful preparation of each thin film (Figure 2). Within the VO_2_ layer (also used as sample S1), the peaks related to lattice motion involving V–V bonds (132 and 229 cm^−1^) as well as the vibrational mode of V–O bonds in the VO_2_ monoclinic insulating phase (500 & 828 cm^−1^) are the most critical [19,20,21,22,23]. For the MoO_3_ thin film, the most robust peaks at 820, 860, and 994 cm^−1^ are related to the α-MoO_3_ crystal phase and the M–O stretching modes [24,25,26]. The weaker peaks at 190, 283, 336, and 352 cm^−1^ are based on Mo–O’s bending mode [27,28], while the one at 658 cm^−1^ is assigned to triply coordinated oxygen Mo_3_–O stretching [29]. Concerning the WO_3_ layer, the monoclinic phase is characterized by peaks at 270 and 326 cm^−1^ [30]. The structure is confirmed via the WO_3_ lattice mode peak at 132 cm^−1^ [31] and the stretching mode peaks of W–O–W bonding at 711 & 810 cm^−1^ [30]. Finally, the Raman peaks for Mo_0.2_W_0.8_O_3_ thin films are very weak. However, the prominent peaks for the WO_3_ are distinguishable, suggesting a severe disruption of the WO_3_ structure with the introduction of Mo. Nevertheless, Raman spectroscopy is not accurate enough for the precision of the structural properties of thin films, which strongly influence their thermochromic performance.

**Figure 3:**
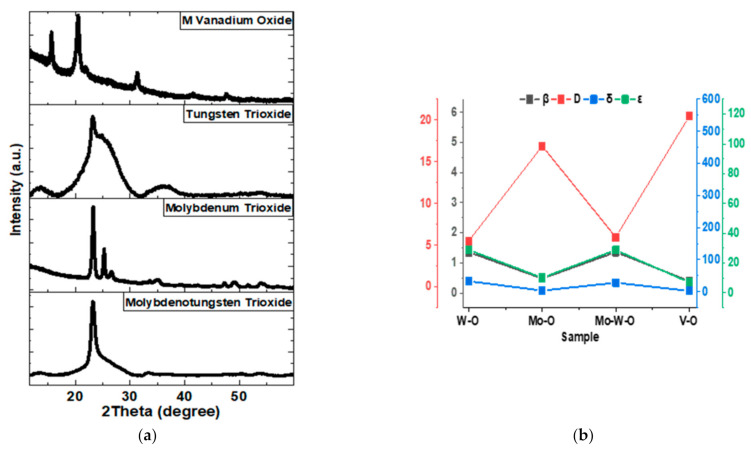
(**a**) XRD spectra of vanadium oxide, tungsten trioxide, molybdenum trioxide, and molybdenotungsten trioxide films. Where (**b**) summarizes the relationship between the FWHM (*β* in (°)), the crystallite size (*D* in (nm)), dislocation density (*δ* in (nm^−2^)), microstrain (*ε* in (unitless) of each layer.

Mo, W, and V metals are metal atoms with large coordination numbers, and the number of phases with oxygen is also large. Some of these phases are metastable, while others are stable. Synthesis conditions such as temperature, oxygen ratio, and deposition pressure are the basic parameters that determine the phase to be formed. The X-ray diffractogram has been used to determine the oxidation state and the structural parameters of the thin films (V–O, W–O, Mo–O, and Mo–W–O) as in Figure 3a, separately. These XRD patterns were obtained by GIXRD examination of thick films (>300 nm) grown on Soda-Lime Glass (SLG) under the same conditions for each layer. The examination was carried out with a grazing angle of 1° and counting for 5 s at each angle value in 0.01° steps. XRD patterns presented separately for each layer were used to determine the phase or phases of the thin film structure. Rietveld structure refinement was performed with the technique described in [17] for Mo–O, and Mo–W–O, W–O phases and the technique described in [32] for the VO_2_ phase, using the FullProf Suite software, which yielded the structure parameters and stoichiometric ratios of metal and oxygen. The use of Rietveld refinement to characterize the structural properties and concentration of vacancies is a frequently used method, reported in numerous reported studies [17,32,33,34,35,36,37]. However, performing structural analysis with XRD patterns that occur when XRD is taken for multilayer structures brings some errors. In the resulting multilayer structure, XRD peaks will occur in a very complex order and it will be difficult to determine which phase they belong to.

Vanadium oxide film shows a monoclinic phase with a space group of C_12_/m1 The diffraction peaks observed at 2*θ* = 27.9, 37.2, 42.3, 55.6, and 57.7° refer to (011), (200), (212), (220), and (022), respectively. The main peak is observed for 2*θ* = 27.9°, indicating a preferential orientation toward the (011) plan. The diffractogram corresponds roughly to the VO_2_ structure. However, the oxygen defects detected via the refinement show the presence of the VO_1.75_ compound. The oxygen vacancies act as electron donors with high n-type conductivity. They can alter the electron orbital occupancy and the band structure of the V–O layer [32,38]. VO_1.75_ layer is a rutile metallic state [39,40,41] which is unstable due to its sensitivity to atmospheric oxygen. Tungsten oxide film shows a monoclinic phase (γ-WO_2.94_) with a space group of P_12_/m1. This phase is also maintained for the Mo–W–O layer (Mo_0.2_W_0.8_O_2.8_). Concerning the molybdenum oxide (MoO_3_), the diffractogram corresponds to an orthorhombic structure with the Pnma space group. However, this layer is biphasic, consisting of 40% MoO_2.08_ and 60% MoO_3.01_. Thus, an oxygen deficiency for each layer has been observed, confirming their metallic characteristic. On the other hand, the XRD patterns of each layer present the central peak at around 23°, which corresponds to the plan (002) of the monoclinic phase and (011) of the orthorhombic structure. The atomic alignment with an angle of 90° for both plans allows overlaying. This characteristic is also true for the plan (011) of the V–O layer. That is why the heterojunction is probably obtained following these orientations within the multilayer structure.

However, the widths of the XRD peaks give us a rough value of the crystallite size with the help of the Debye–Scherrer equation. The crystallite size was calculated from the Debye–Scherrer equation (*D* = 0.9*λ*/*β*cos*θ*). After that, the main peak positions (2*θ*) and their corresponding FWHM (*β*), crystalline size (*D*), dislocation density (*δ*, where *δ* = 1/*D*^2^), and microstrain (*ε*, where *ε* = *β*/4tan*θ*) were estimated (Table 2). Although this value is not exact, it is valuable in terms of speculating. Decreased peak widths indicate an increase in crystallite size. It is important to note that the XRD of WO_3_ shows crystalline and amorphous phases together. As the growth temperature of the W–O film is 400 °C, this temperature is close to the W–O crystallization temperature. The W–O structure crystallizes in the range of 400–600 °C [42].

In order to calculate the FWHM for the crystallite size in the structures containing W-O, a deconvolution was performed using the PeakFit software (Version 4.12), assuming a Lorentzian-shaped peak located at about 23 degrees. The crystallite size calculated for Mo-O was found to be larger than the W–O film. It was also found to be relatively small for the Mo–W–O layer. Penetration of Mo into the W–O layer resulted in a reduction of the main peak width. With the incorporation of Mo atoms into the W–O structure, an expected increase in the crystallite size, hence in the crystallinity, occurred, and it can be said that W–O crystal structures is maintained even in the presence of Mo atoms. Thus, the crystallinity of the oxide layers is improved with the presence of Mo in contrarily to W [43]. The calculation shows that the crystallite size for the W–O film (5.5 nm) is much smaller than for Mo–O (17.0 nm), while the crystallite size for Mo–W–O is intermediate (6.0 nm). However, the largest crystallites were calculated for the V–O layer with 20.5 nm. The evolution of the crystallite size values is inversely proportional to the evolution of dislocation density and microstrain. In other words, small crystallite size, high dislocation density, and high microstrain were calculated for W–O thin film. In contrast, the opposite was calculated for Mo–O and V–O layers. Nevertheless, all values are low for Mo–W–O thin film, suggesting that the Mo doping can add new properties to the W–O-based thin film.

### 3.2. Effect of the Buffer Layer on the VO_2_ Properties

The MIT properties of the multi-layer oxide structure are governed via the behaviour of the VO_2_ layer. As seen above, the buffer layer (WO_3_ and MoO_3_) effect on vanadium-based oxide has been studied (S1–S3). Raman spectroscopy analysis of these structures S2–S6 is depicted in Figure S1 (supplementary information). Similar peaks are observed for these structures, as have been reported above for the single layers. The topographical analysis of MoO_3_ and WO_3_ deposited only on the glass substrate is investigated as in Figure S2.

**Figure 4:**
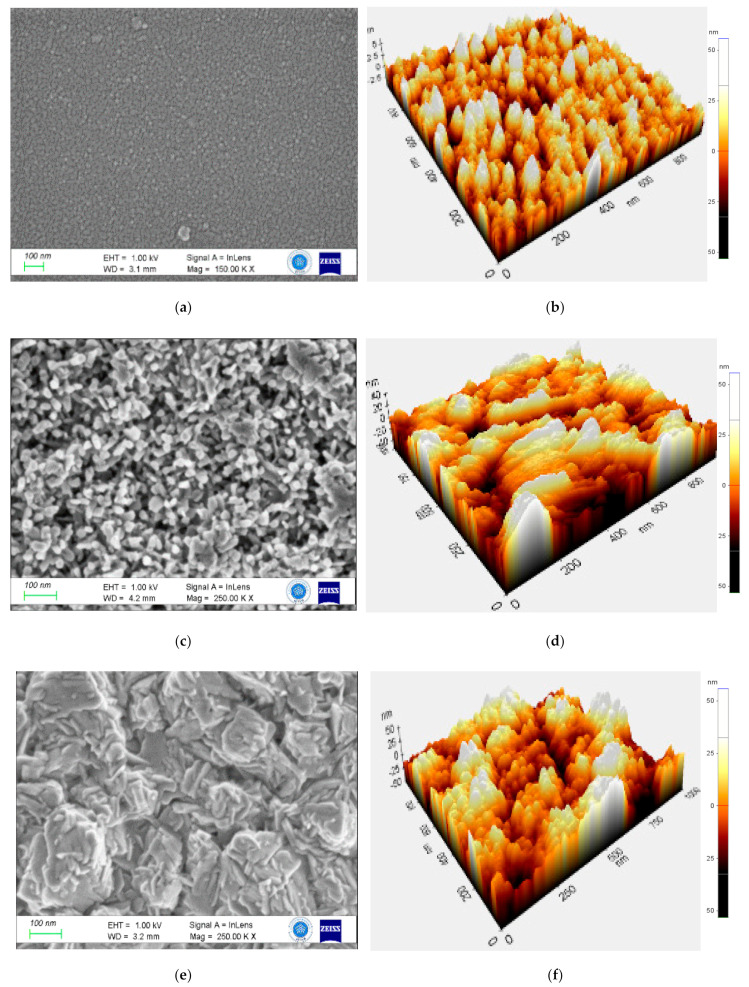
Morphological and topographical analysis of S1 (**a**,**b**), S2 (**c**,**d**), and S3 (**e**,**f**) samples, respectively.

First of all, the morphology of the VO_2_ thin film is strongly influenced by the buffer layer (Figure 4). The VO_2_ layer, as deposited on the FTO glass (S1), consists of small particles (about 20 nm as predicted with XRD) homogeneously and densely distributed. Table 3 provided information about the topographic data of S1–S6 samples. The mean grain area is below 9.5 × 10^−3^ μm^2^, involving a very low average roughness (Ra). However, when deposited on the MoO_3_ layer (S2), VO_2_ grains are bigger, and aggregates are visible on the surface. The average roughness is more than 12 times bigger than for S1, according to the relief observed on the FESEM picture. Concerning the WO_3_ bottom layer (S3), small particles completely disappear, and clusters with nano leaves are present on the surface of the VO_2_ thin film. Even if the mean grain area reaches more than 1.77 × 10^−3^ μm^2^, Ra values are only about 6 nm. This effect has been ascribed to the monoclinic crystal structure of WO_3_ film, which favours electron diffusion [44]. Finally, these three samples confirm that the VO_2_ thin film morphology is sensitive to any internal or external strain or stimuli [45,46].

Used as support for metal oxide layers, it is known that FTO glass is transparent at more than 70% on the UV-visible-NIR domains. However, the optical properties are different according to the metal oxide layers (Figure S3). The transparency is reducing below 40% for the VO_2_ layer. However, for the Mo–O, Mo–W–O, and W–O layers, the transparency can reach values higher than 80% up to almost 100% Mo-W–O at around 500 nm.

**Figure 5:**
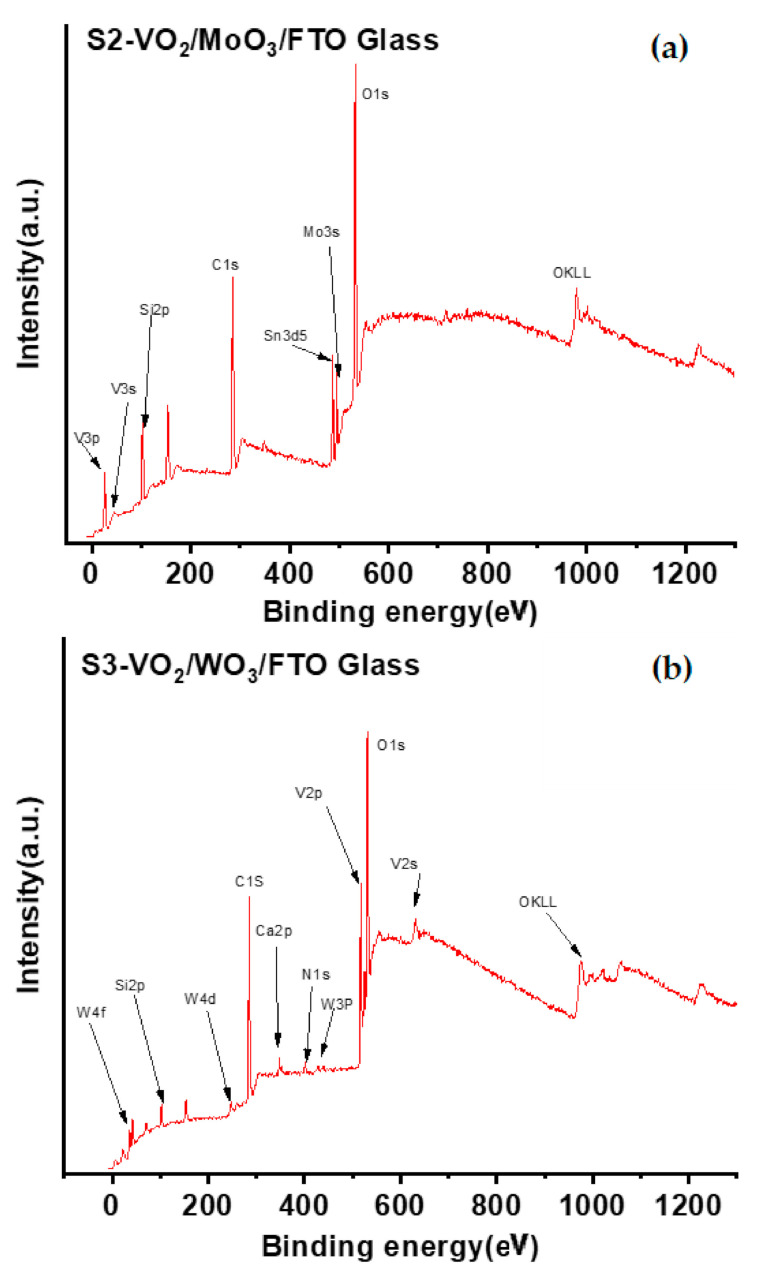
Fast XPS scan of some selected samples: (**a**) S2 and (**b**) S3.

On the other hand, the surface bonding is also affected by the effect of the buffer layer. The vanadium binding on the XPS spectrum of the film supported with WO_3_ (S3) is characteristic of highly oxidized vanadium (V^4+^/V^5+^) at around 517 and 524 eV [47,48,49]. While peaks are related to low oxidization (V^3+^) at around 41 and 68 eV when supported with MoO_3_ (S2) (Figure 5a,b) [50,51]. In other words, the interaction between molybdenum and vanadium oxides allows for keeping an oxidation degree below 4. In contrast, the vanadium-based layer is more sensitive to oxidation when supported with WO_3_. Therefore, MoO_3_ has been preferred as a buffer layer for the samples aiming to study the anti-reflective top layer effect on the thermochromic properties of vanadium oxide. In addition, the observed signals of Na, Ca, Sn, and Si stem from the thin layer of the FTO glass substrate. It is important to mention that all films were deposited with high vacuum sputtering techniques, with ultrahigh homogenous depositions. These conditions avoid the absence of covering some parts during the deposition of S2. The diffusion of some elements from the substrate into the S2 is highly expected during the film deposition. This is because the films were deposited at an approximately high temperature of 400 °C, followed by an in situ annealing at 400 °C for around 2 h. These two processes may cause direct diffusion of some elements from the substrate into the film.

### 3.3. Anti-Reflective Top Layer Surface State

**Figure 6:**
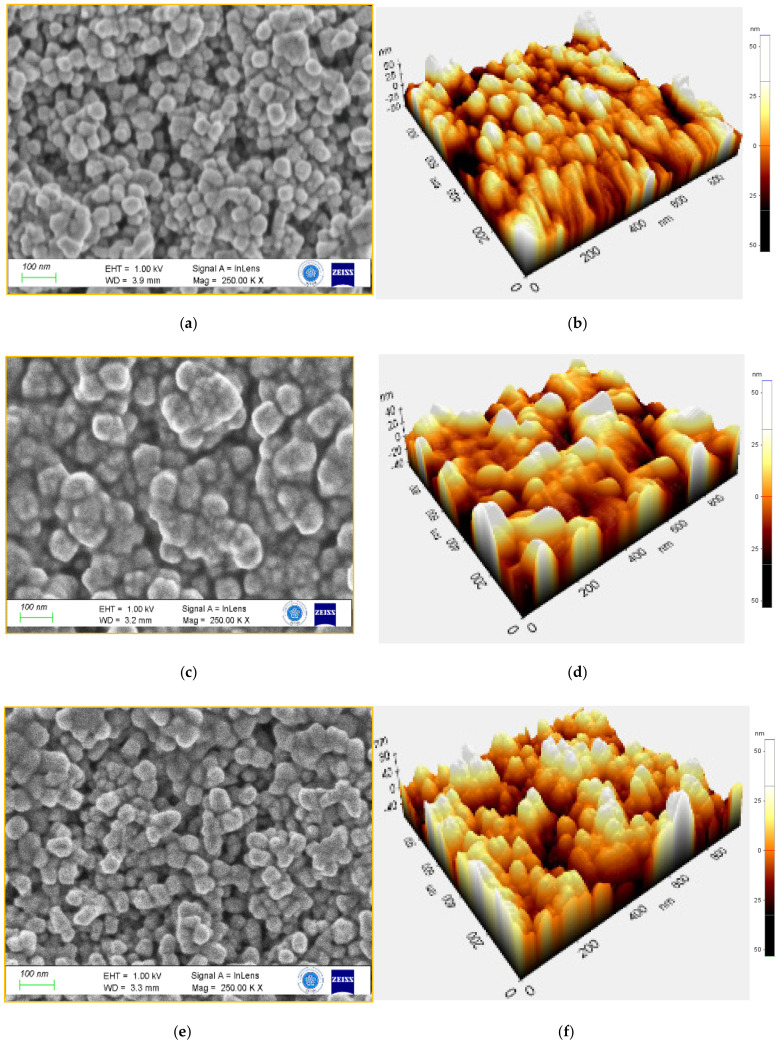
Morphological and Topographical analysis of S4 (**a**,**b**), S5 (**c**,**d**), and S6 (**e**,**f**) samples.

Due to their optical properties of WO_3_ (S4) and Mo_0.2_W_0.8_O_3_ (S5) were coated in situ on the surface of MoO_3_ supported VO_2_ layer. Both surfaces contain particles that locally aggregate, inducing pores formation between them (Figure 6). The topographic data show that the surface roughness of S4 is comparable to S2. The WO_3_ top layer can fit the structure of the VO_2_ thin film. However, when 20% W is substituted with Mo within the top layer (S5), its particle’s grain area increases from 1.183 × 10^−3^ to 1.526 × 10^−3^ μm^2^, involving lower surface roughness. The former presents smaller particles and higher roughness when deposited on W doping of the VO_2_ (S6). The multilayer morphological and topographical analysis demonstrates that the surface is dependent mainly on the bottom layer state and, to a lesser extent, on the upper layer properties.

### 3.4. FTIR Spectroscopic, Optical Properties, and Urbach Tail

Here, we investigated the optical properties of the deposited thin film with UV-visible-NIR spectroscopy and FTIR spectroscopy, as depicted in Figure S4 (supplementary information). The FTIR spectrum of S1 presents three prominent absorption peaks at around 400, 766, and 901 cm^−1^ (Figure S4a). Even if they are attributed to the V-O stretching mode for the lowest wavenumber [52] and lattice vibration mode for the two others [53,54], these peaks are not Gaussian. However, the UV-visible-NIR spectroscopy shows the optical transmission as in Figure S4b. Introducing W into the VO_2_ structure improves the transparency close to the support values. This evolution can probably be explained by two reasons, the electron density evolution of the local crystal strain introduction within the VO_2_ layer.

The calculation of the Urbach energy (*E_U_*) evolution provides a clue concerning the transmittance fall observed (Figure S5a,b). *E_U_* is visible in low crystalline, disordered, or amorphous materials with localized states in the standard bandgap due to exciton/phonon or electron/phonon interactions. The results show that with the modification of the VO_2_ structure, the electronic transitions from band to tail and tail to tail are facilitated with probably a better order between the layers [55]. Thus, the combination Mo_0.2_W_0.8_O_3_/W-VO_2_/MoO_3_ is optimum regarding the optical properties of the present materials.

### 3.5. Electric Properties

**Figure 7:**
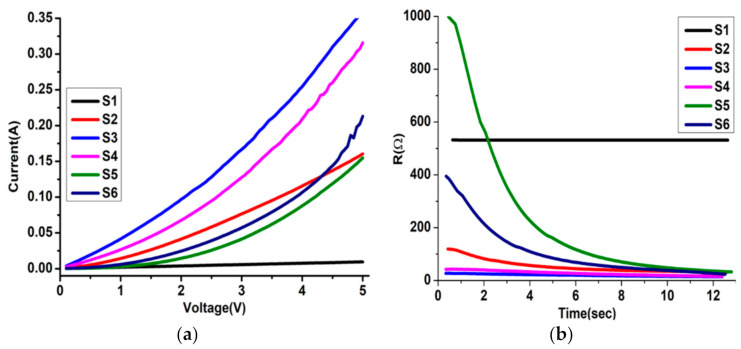
(**a**) The characteristic I–V curves of S1–S6 thin films and (**b**) the corresponding change in R-t curves of the measured I–V curves of each film.

Due to the specificity of the correlated oxide, the evolution of the structural, optical, and electrical properties is parallel with temperature. Thus, following the electrical properties is an alternative to understanding the MIT characteristics. An Ohmic behaviour is observed at room temperature for S1 and S2. In contrast, a Schottky contact is detected for the others (S3 to S6) (Figure 7a). However, the most resistive sample is S1, and the most conductive are S3 and S4, which are the only ones containing the WO_3_ layer. More than the samples’ multilayer and grain structure, the influence of the intrinsic conductivity of WO_3_ is the most important parameter [56]. As seen above, WO_3_ favours the formation of V_2_O_5,_ which is the highest oxidative state among the vanadium oxides and the most resistive [57]. On the other hand, the presence of WO_3_ and/or MoO_3_ layers does not significantly affect samples’ response time, contrarily to the samples (S5 and S6) where the Mo_0.2_W_0.8_O_3_ layer is involved (Figure 7b). Whatever the combination, after roughly 9 s, the resistance of multilayer samples reaches the low values. Since Mo_0.2_W_0.8_O_3_ contains similarly WO_3_, MoO_2_, and MoO_3_ phases, and electrons diffusion are probably deleted within this layer. Finally, except for S1 which is purely resistive, the other samples present a semi-conductive behaviour.

**Figure 8:**
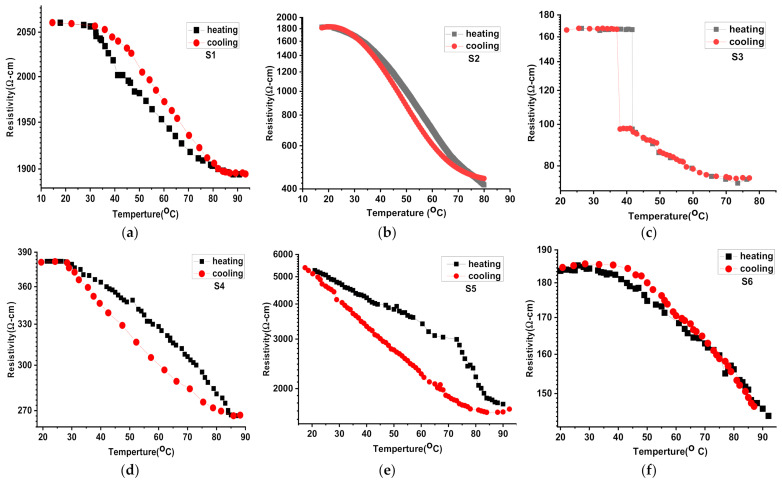
Electric properties and phase transition characterization measurements of the prepared S1–S6 (**a**–**f**) thin films.

The VO_2_ phase change from monoclinic to tetragonal crystal structure with temperature involves the decrease of the resistivity. Therefore, the MIT value (also denoted T_c_) for each structure can be evaluated by following the electrical properties of the samples. When a sharp decrease is not observed in the resistivity, the measured MIT will be considered from the starting point of the decrease. In this work, the MIT for all samples is lower than the theoretical value T_c_ = 68 °C for VO_2_ (Figure 8a,f). According to the characterization above, the typical profile for the VO_2_ (VO_1.73_) layer is observed for S1. The low MIT at about 35 °C is due to the oxygen vacancies [58,59].

Nevertheless, the high resistivity values and the hysteresis observed between the heating and cooling curves show a forced transition phase. When deposited on MoO_3_ (S2), the VO_2_ phase transition starts at lower T_c_ (25 °C), and the hysteresis dramatically disappears. The strain effect of MoO_3_ combined with the oxygen deficiency is profitable; however, the resistivity is still high. The resistivity is significantly reduced when WO_3_ is a bottom layer (S3), and a sharp resistivity decrease appears at 41 °C during heating. The T_c_ is higher than for S1, but the theoretical abrupt decrease is only visible with this sample.

On the other hand, the lowest resistivity value is not reached at 41 °C, and the XPS results from the formation of V_2_O_5_ at the film surface. Therefore, it is suggested that WO3 contributes to improving the structure’s conductivity. However, probably due to its insufficient strain effect, it cannot prevent the oxidation of VO_2_ to V_2_O_5_, the most stable vanadium oxide. Currently, WO_3_ is used as an anti-reflective layer. Therefore, sandwich structure with MoO_3_ at the bottom and WO_3_ at the upper layer was investigated with S4. As expected, the MIT was reduced to T_c_ = 30 °C, and the resistivity values were low. Unfortunately, a critical hysteresis appears. Since the presence of Mo seems to facilitate the strain effect and based on the performance demonstrated within our previous work, Mo_0.2_W_0.8_O_3_, which is a Mo doped WO_3_ thin film, was tested as the upper layer (S5). The lower MIT temperature (about 20 °C) and the highest resistivity values are reached, and a critical hysteresis is observed with S5. The introduction of Mo within the WO_3_ structure involves the simultaneous presence of MoO_2_, MoO_3,_ and WO_3_. The presence of MoO_3_ possibly contributes to the strain of the VO_2_ structure. However, it also disrupts the WO_3_ continuity involving a drop in the conductivity. Due to its confirmed anti-reflective and anti-oxidative performance, Mo_0.2_W_0.8_O_3_ was kept as the upper layer. W-doped VO_2_ was designed to decrease the sample S6 resistivity. The W doping is exceedingly profitable in decreasing the hysteresis and the resistivity of the structure. However, T_c_ increases to up to 36 °C. Therefore, thanks to the sandwich structure prepared with two doped thin films, the hysteresis and the resistivity decreased significantly by almost keeping the reference T_c_. Based on the literature reviews, we conclude that our study is a facile and efficient way to modulate the phase transition of VO_2_ thin film as in Table 4.

**Table 4:** Summary of the previous studies and the advantages of our current study.

Structure	Preparation Method	Tunneling Effect	Phase Transition Temperature	Ref.
Cr-doped VO_2_	Pulsed laser deposition	Doping	34 °C	[57]
Mo-doped VO_2_	DC sputtering	Doping	63 °C	[58]
W-doped VO_2_	Sol–gel	Doping	36 °C	[47]
W-doped VO_2_	DC sputtering	Doping	37.4 °C	[59]
Al-doped VO_2_	DC sputtering	Doping	44.9 °C	[60]
V_2_O_5_/metal V/V_2_O_5_, V_2_O_5_/metal W/V_2_O_5_ multilayers	RF sputtering	Sandwich structure	55 °C 48 °C	[61]
VO_2_ on MgF_2_ (110)	Oxide MBE method	Interfacial strain and oxygen vacancies	69 °C	[62]
W-doped VO_2_	Hydrothermal	Doping	31.64 °C	[63]
VO_2_	Sol–gel dip coating	-	68 °C	[64]
Mo_0.2_W_0.8_O_3_/VO_2_/MoO_3_	DC, RF Sputtering	Oxygen vacancy concentration, lattice strain, and W-doping effects	20 °C	This work

## 4. Conclusions

VO_2_ is one of the most investigated phase transition materials since the MIT is observed close to ambient temperature. According to the application, setting the T_c_ is a crucial parameter. In this study, the MIT temperature decreased to 41 °C via oxygen-vacancy vanadium dioxide (VO_1.73_) through the deposition conditions. The evolution of the bandgap and the Urbach tail demonstrate the influence of the bottom and top layers on the VO_2_ layer. These interactions are probably guided by the common vibration observed in the Raman spectrum between two layers which have an interface. The results showed that the structure of the VO_2_ layer is more preserved when MoO_3_ is used than WO_3_. The electrical measurements show that the structure resistance is lower, and the phase transition is easier with the WO_3_ bottom layer. On the other hand, WO_3_ does not reduce the hysteresis between the heating and cooling curve contrarily to MoO_3_. The former decreases the T_c_ down to 25 °C, and the hysteresis is significantly reduced. Therefore, the combination of MoO_3_ bottom layer and WO_3_ upper layer was investigated with the WO_3_/VO_2_/MoO_3_, in which resistance is low, T_c_ of VO_2_ is kept, but the hysteresis is still present. Meanwhile, the high microstrain on VO_1.75_ film of 119.9 × 10^−3^ is exerted by the monoclinic WO_2.94_ buffer layer, which contributed to a fast and sharp phase transition WO_2.94_/VO_1.75_/FTO glass with a low phase transition. The transmittance of Mo-doped WO_3_ (Mo_0.2_W_0.8_O_3_) is higher than WO_3,_ and the dislocation density is comparable to the VO_2_ layer. The potential of Mo_0.2_W_0.8_O_3_ as an alternative upper layer was confirmed with Mo_0.2_W_0.8_O_3_/VO_2_/MoO_3_, where the Tc decreases to 20 °C. Nonetheless, the hysteresis and the resistance are incredibly increasing. To maintain the profit of Mo_0.2_W_0.8_O_3_ and improve the conductivity of the sample, VO_2_ was doped with metallic W. The combination Mo_0.2_W_0.8_O_3_/VO_2_–W/MoO_3_ successfully showed low T_c_ at 36 °C, low resistivity at tens of ohm, and no hysteresis. Thanks to this study, the synergy between oxygen deficiency, strain engineering, and metal doping was reached for improving the MIT characteristics of VO_2_ thin film.

## Figures and Tables

**Table 1:** Abbreviation of samples and deposition parameters of the multilayer films.

Notation	Structure	Layer	Power of Mo (W) RF1	Power of W (W) RF2	Power of V (W) DC	Ar Flow (sccm)	O_2_ Flow (sccm)	Time (min)
S1	VO_2_	1st layer: VO_2_	-	-	190	41	2.2	7.5
S2	VO_2_/MoO_3_	1st layer: MoO_3_	137	-	-	37.1	12.1	16.7
		2nd layer: VO_2_	-	-	190	41	2.2	7.5
S3	VO_2_/WO_3_	1st layer: WO_3_	-	137	-	37.1	12.1	7.5
		2nd layer: VO_2_	-	-	190	41	2.2	7.5
S4	WO_3_/VO_2_/MoO_3_	1st layer: MoO_3_	137	-	-	37.1	12.1	16.7
		2nd layer: VO_2_	-	-	190	41	2.2	7.5
		3rd layer: WO_3_	-	137	-	37.1	12.1	7.5
S5	Mo_0.2_W_0.8_O_3_/VO_2_/MoO_3_	1st layer: MoO_3_	137	-	-	37.1	12.1	16.7
		2nd layer: VO_2_	-	-	190	41	2.2	7.5
		3rd layer: Mo_0.2_W_0.8_O_3_	27	110	-	37.1	12.1	7.5
S6	Mo_0.2_W_0.8_O_3_/VO_2_ + W/MoO_3_	1st layer: MoO_3_	137	-	-	37.1	12.1	16.7
		2nd layer: VO_2_ + W	-	10	190	41	2.2	7.5
		3rd layer: Mo_0.2_W_0.8_O_3_	27	110	-	37.1	12.1	7.5

**Table 2:** Shows the main peak position, corresponding FWHM, crystallite size, dislocation density, and microstrain of the samples.

Sample	2*θ* (°)	*β* (°)	*D* (nm)	*δ* × 10^−3^ (nm^−2^)	*ε* × 10^−3^
V–O	27.936	0.399	20.5	2.4	7.01
Mo–O	23.254	0.479	17.0	3.5	10.18
Mo–W–O	23.309	1.359	6.0	28.1	28.76
W–O	23.222	1.358	5.5	32.9	28.84

**Table 3:** Topographic data of S1–S6 samples.

Sample	Mean Grain Area (μm^2^)	R_a_ (nm)	R_q_ (nm)	R_pv_ (nm)
S1	9.41 × 10^−4^	0.9	1.2	6.8
S2	1.183 × 10^−3^	12.2	14.1	56.1
S3	1.774 × 10^−3^	6.0	7.4	33.5
S4	1.056 × 10^−3^	10.1	12.1	49.9
S5	1.526 × 10^−3^	5.7	6.9	28.1
S6	9.06 × 10^−4^	13.8	16.1	63.1

R_a_: Average roughness, R_q_: root mean square or standard deviation of the height value, and R_pv_: height difference or peak-to-valley.

## Data Availability

The data presented in this study are available on request from the corresponding author.

## Supplementary Materials

The following supporting information can be downloaded at: https://www.mdpi.com/article/10.3390/nano12091470/s1, Figure S1: Raman spectra of the multi-layers-based phase transition devices. Figure S2: Topographical analysis of MoO_3_ (a) and WO_3_ (b). Figure S3: Transmission spectra (T) of the single-layer thin films. Figure S4: (a) FTIR spectra of samples S1–S6 at room temperature. (b) UV-Vis-NIR spectra of samples S1–S6 at room temperature. (c,d) the refractive index and extinction coefficient of S1–S6 thin films with wavelengths. Figure S5: (a) and (b) represent the plot of (Ln α) versus photon energy and the Urbach energy (Urbach tail) with bandgap for S1–S6 thin films, respectively. References [65,66,67,68,69] are cited in the supplementary materials.
