# Supplementary material for: Tuning the Metal–Insulator Transition Properties of VO2 Thin Films with the Synergetic Combination of Oxygen Vacancies, Strain Engineering, and Tungsten Doping
Source: Nanomaterials (Basel). 2022 Apr 26;12(9):1470. doi: 10.3390/nano12091470 (PMC9099983; doi:10.3390/nano12091470)
Supplement: Supplementary file 1 [file nanomaterials-12-01470-s001.zip › nanomaterials-1650049-supplementary.pdf]

Supplementary Information

Tuning the Metal–Insulator Transition Properties of VO_2 Thin Films with the Synergetic Combination of Oxygen Vacancies, Strain Engineering, and Tungsten Doping

Mohamed A. Basyooni [1,2,3], Mawaheb Al-Dossari [4,5], Shrouk E. Zaki [1,3], Yasin Ramazan Eker [3,6,*], Mucahit Yilmaz [2] and Mohamed Shaban [7,8,*]

1 Department of Nanotechnology and Advanced Materials, Graduate School of Applied and Natural Science, Selçuk University, Konya 42030, Turkey; m.a.basyooni@gmail.com (M.A.B.); shrouk.e.zaki@gmail.com (S.E.Z.)
2 Department of Nanoscience and Nanoengineering, Institute of Science and Technology, University of Necmettin Erbakan, Konya 42060, Turkey; mucahityilmaz@erbakan.edu.tr
3 Science and Technology Research and Application Center (BITAM), University of Necmettin Erbakan, Konya 42060, Turkey
4 Research Center for Advanced Materials Science (RCAMS), King Khalid University, Abha 61413, Saudi Arabia; mdosri@kku.edu.sa
5 Department of Physics, Dhahran Aljanoub, King Khalid University, Abha 61421, Saudi Arabia
6 Department of Metallurgy and Material Engineering, Faculty of Engineering and Architecture, Necmettin Erbakan University, Konya 42060, Turkey
7 Department of Physics, Faculty of Science, Islamic University of Madinah, AlMadinah Almonawara 42351, Saudi Arabia
8 Nanophotonics and Applications Laboratory, Physics Department, Faculty of Science, Beni-Suef University, Beni-Suef 62514, Egypt
* Correspondence: yeker@erbakan.edu.tr (Y.R.E.); mssfadel@aucegypt.edu (M.S.)

Effect of the buffer layer on the VO_2 properties

Raman spectroscopy analysis of S2–S6 is depicted in Figure S1. Similar peaks are observed for these structures, as have been reported above for the single layers.

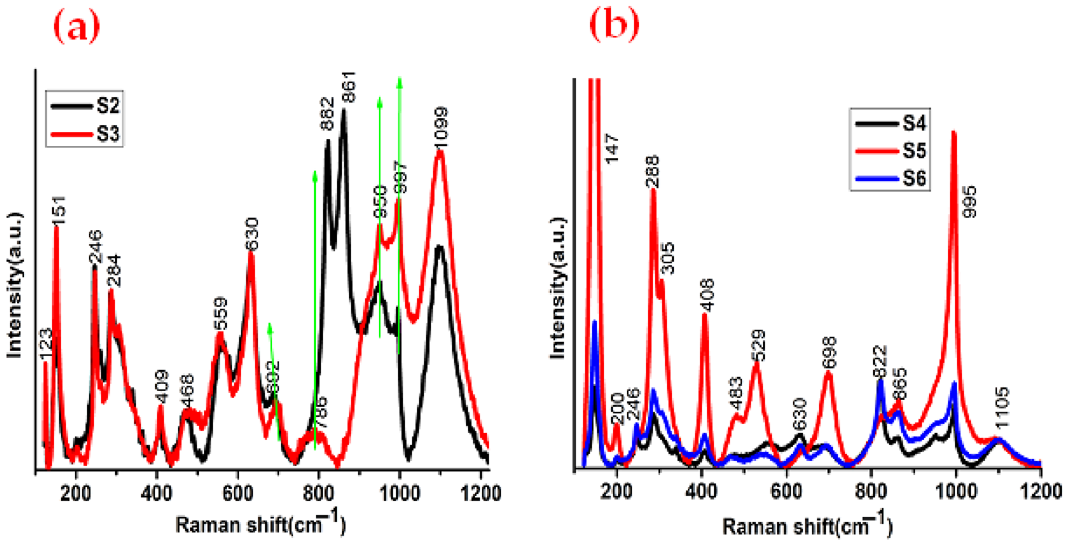

Figure S1. Raman spectra of the multi-layers-based phase transition devices. (a) Raman peaks of S2 and S3, (b) Raman peaks of S4, S5, and S6 at RT.

The topographical analysis of MoO_3 and WO_3 deposited only on the glass substrate is investigated as in Figure S2. We see that the topography of the MoO_3 surface is highly rough with bigger surface particles. While for the case of WO_3, the surface become fine with lower roughness than the MoO_3 surface.

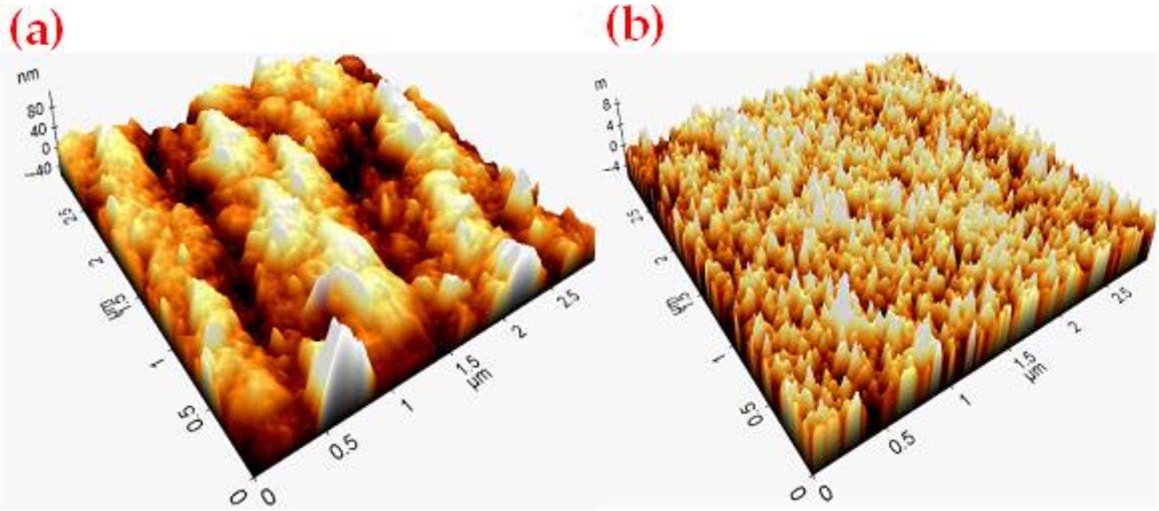

Figure S2. Topographical analysis of MoO_3 (**a**) and WO_3 (**b**).

Spectroscopic and Optical properties

Used as support for metal oxide layers, It Is known that FTO glass is transparent at more than 70% on the UV-visible-NIR domains. However, the optical properties are different according to the metal oxide layers (Figure S3). The transparency is reducing below 40% for the VO_2 layer. However, for the Mo–O, Mo–W–O, and W–O layers, the transparency can reach values higher than 80% up to almost 100% Mo–W–O at around 500 nm. These results demonstrate an interaction between FTO and metal oxide layers. In this perspective, as predicted in strain engineering principles, the properties of samples prepared with several oxide layers will change according to the origin of the top, bottom, and intermediate layers. Despite the decimal stoichiometries, to facilitate the reading, VO_2, MoO_3, WO_3 and $Mo_{0.2}W_{0.8}O_3$ have been conserved along with the text.

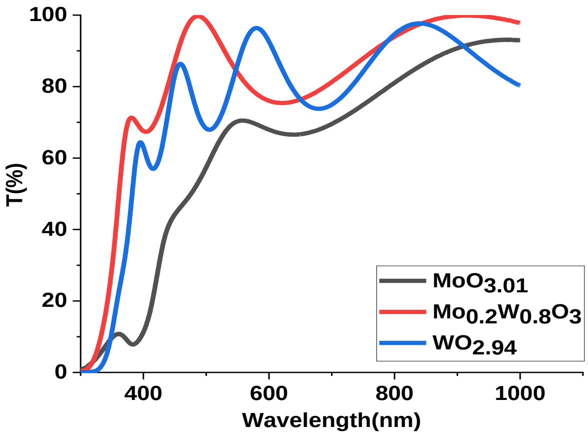

Figure S3. Transmission spectra (T) of the single-layer thin films.

Especially for radiative cooling (thermochromic) applications, the optical properties of the samples have been investigated at wavelengths from visible to infrared domains. The goal is to have high transmittance on both domains at room temperature after heating above MIT to keep transparency on a visible domain and stop it on the infrared domain. Typically, the vibrational mode is excited, involving specific absorption peaks relative to atomic bonds. The absorption peaks of metal oxides are peculiarly present at wavenumbers below 1000 cm^{-1} as in Figure S3a. The oxide bonds for the sample S1 are at least Si–O (from glass support), Sn–O (from FTO coating), and V–O (from the deposition). The FTIR spectrum of S1 presents three prominent absorption peaks at around 400, 766, and 901 cm^{-1} (Figure S3a). Even if they are attributed to the V–O stretching mode for the lowest wavenumber [1] and lattice vibration mode for the two others [2,3], these peaks are not

Gaussian. Thus they are probably a combination of several IR modes found with the deconvolution process (not helpful for this work).

On the other hand, the transmittance reaches a maximum at about 1250 cm^{-1} and progressively decreases above this wavenumber up to 4000 cm^{-1}. Concerning the S2 to S6, the FTIR spectrum profile is similar to the S1 for wavenumbers below 1250 cm^{-1}. Nevertheless, at higher values, the transmittance increases vigorously and continuously. These results show that WO_3, MoO_3, and $Mo_{0.2}W_{0.8}O_3$ thin films present in the multi-layer structure affect the VO_2 properties to give the ability to transmit the infrared rays, which are responsible for radiative heating.

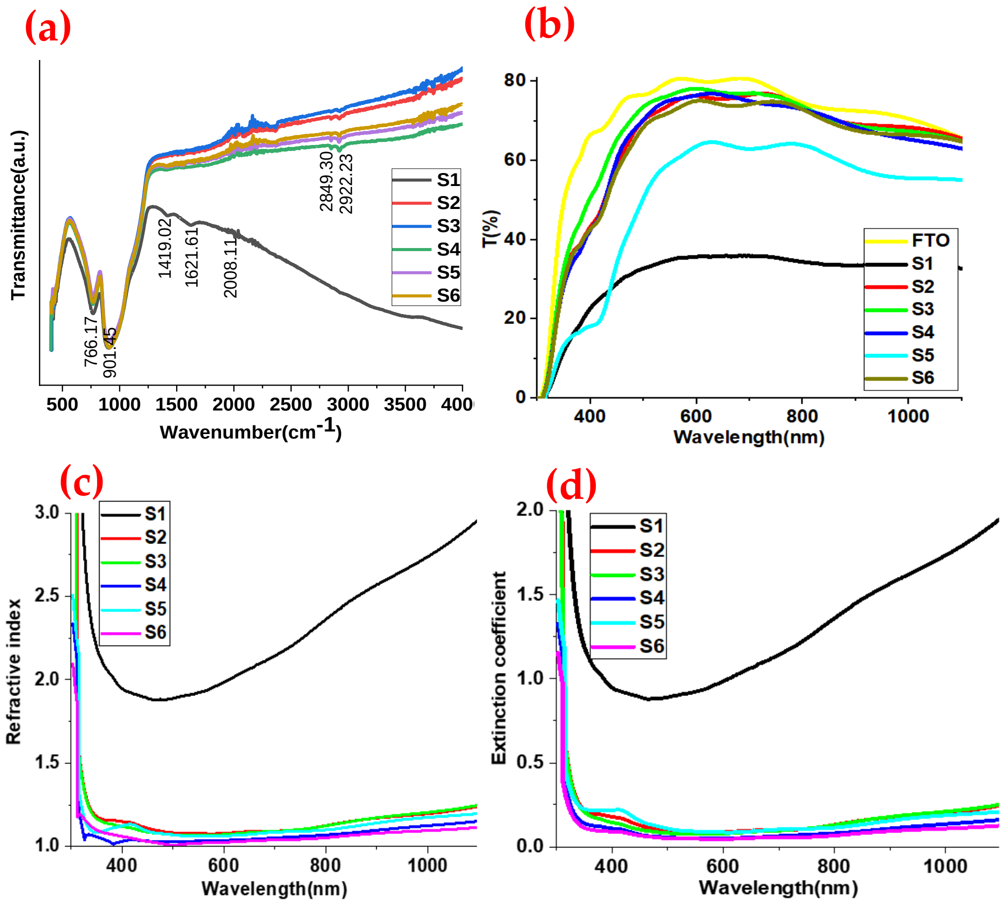

Figure S4. (**a**) FTIR spectra of samples S1–S6 at room temperature. (**b**) UV-Vis-NIR spectra of samples S1–S6 at room temperature. (**c**,**d**) the refractive index and extinction coefficient of S1–S6 thin films with wavelengths.

Subsequently, the transmittance evolution of the multi-layer structures has been investigated in the range of 300–1000 nm (UV-vis-NIR) (Figure S4b). The FTO coated glass has the highest transmittance at about 80%, which falls to about 30% with the VO_2 coating. However, due to the strain effect of the WO_3 or MoO_3 layers, the transmittance of VO_2 can reach values close to the support one. On the other hand, the transmittance at about 400 nm is strongly dependent on the presence of Mo in the sample since only S1 and S3 do not show a fall down at this wavelength. As for $Mo_{0.2}W_{0.8}O_3$, which is applied as an anti-reflective layer for the first time, the transmittance is reduced to about 60% (S5). However, the introduction of W into the VO_2 structure improves the transparency close to the support values. This evolution can probably be explained by two reasons, the electron density evolution of the local crystal strain introduction within the VO_2 layer.

Furthermore, by using the reflectance measurements, the refractive index (n) [4–6] and the extinction coefficient ($k = \alpha\lambda/4\pi$) in function with wavelength have been calculated (Figure S4c,d). Both values present the same evolution for all samples, a sharp decrease below 400 nm (UV domain) after a small increase (visible and near-infrared domain).

However, the increase above 400 nm is more pronounced for S1 than the other samples. Moreover, except for S1 above 330 nm, n values are weak below 1.1 while *k* values are below 0.25. S6 presents the lowest values which are *n* = 1 and *k* = 0.05 at 500 nm. Regarding S1, *n* > 1.75 and *k* > 0.80 indicate the FTO's opacity supported VO_2 on the whole domain. Finally, even if its transmittance is at an intermediate level between S1 and the others, *n* and *k* values of S5 are also low, inducing the reflectance of $Mo_{0.2}W_{0.8}O_3$ deposited on the VO_2 layer is greater than WO_3 on VO_2.

Urbach tail calculations

The calculation of the Urbach energy evolution gives a clue concerning the transmittance fall observed with S5 (Figure S5a,b). The Urbach tail is the exponential part along the absorption coefficient curve and near the optical band edge. The inverse of the logarithmic slope gives the Urbach energy ($\ln\alpha = \ln\alpha_0 + (h\upsilon/Eu)$ where α: absorption coefficient, α_0: sample dependent constant, hυ: incident photon energy, *Eu*: Urbach Energy). *Eu* is visible in low crystalline, disordered or amorphous materials with localized states in the standard bandgap due to exciton/phonon or electron/phonon interactions. Therefore the disorder evolution of the phonon state is proportional to the evolution of the *Eu* [7,8]. For S1 to S6 samples, the optical band gap changes between 3.55 to 3.90 eV, while the Urbach energy varies from 1 to 3.5 eV. The multi-layer structure with different oxides as well as their thickness is the reason for these critical differences. Considering the evolution domains of bandgap and EU, S1 and S2 have both low bandgap and low *Eu*. In other words, the MoO_3 bottom layer does not strongly influence the VO_2 structure.

Regarding S3 and S4, the presence of WO_3 as the bottom or upper layer mainly affects the electronic properties increasing the bandgap at about 3.85 eV. By introducing Mo within the WO_3 structure (S5), the bandgap can decrease to 3.6 eV. However, in this case, the disorder increases to give the highest *Eu* value among the present samples [9]. Finally, the W doping of the VO_2 intermediate layer involves the reduction of the *Eu* by keeping the bandgap below 3.6 eV. This means that with the modification of the VO_2 structure, the electronic transitions from band to tail and tail to tail are facilitated with probably a better order between the layers [10]. Thus, the combination $Mo_{0.2}W_{0.8}O_3$/W–VO_2/MoO_3 is optimum regarding the optical properties of the present materials.

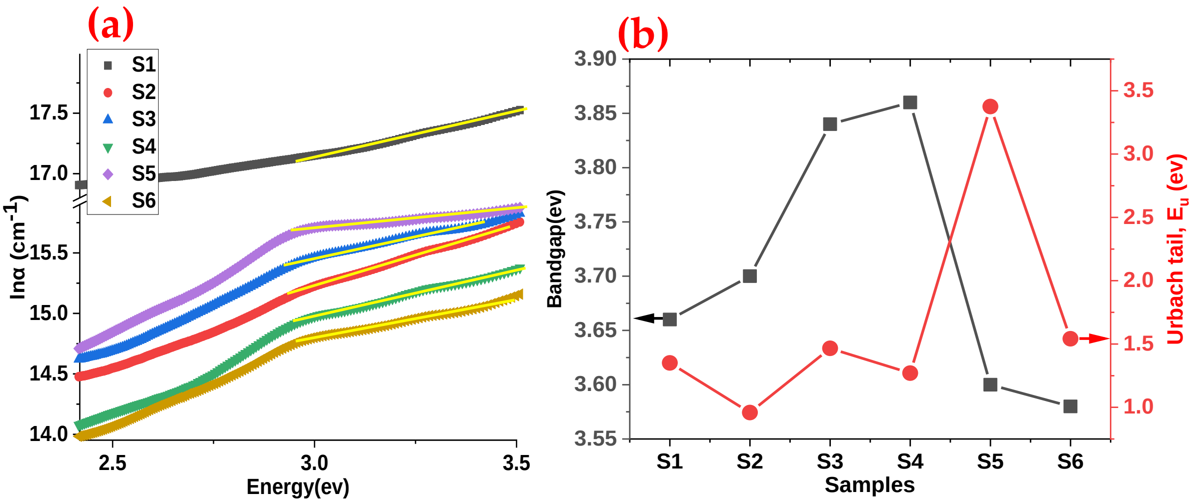

Figure S5. (a) and (b) represent the plot of (Ln α) versus photon energy and the Urbach energy (Urbach tail) with bandgap for S1–S6 thin films, respectively.

References

1. Luo, Z.; Wu, Z.; Xu, X.; Wang, T.; Jiang, Y. Electrical and optical properties of nanostructured VO_x thin films prepared by direct current magnetron reactive sputtering and post-annealing in oxygen. *Thin Solid Films* **2011**, *519*, 6203–6207. https://doi.org/10.1016/j.tsf.2011.03.003.
2. Nam, S.P.; Noh, H.J.; Lee, S.G.; Lee, Y.H. Electrical properties of vanadium tungsten oxide thin films. *Mater. Res. Bull.* **2010**, *45*, 291–294. https://doi.org/10.1016/j.materresbull.2009.12.028.

3. Vedeanu, N.; Cozar, O.; Stanescu, R.; Cozar, I.B.; Ardelean, I. Structural investigation of new vanadium-bismuth-phosphate glasses by IR and ESR spectroscopy. In Proceedings of the Journal of Molecular Structure; 2013; Vol. 1044, pp. 323–327.
4. Görmez, A.E.; Basyooni, M.A.; Zaki, S.E.; Eker, Y.R.; Sönmez, E.; Yılmaz, M. Effect of in-/ex-situ annealing tem-perture on the optical, structural and gas sensing dynamics of CdS nanostructured thin films. *Superlattices Microstruct.* **2020**, *142*, 106536. https://doi.org/10.1016/j.spmi.2020.106536.
5. Mohamed, B.; Ali, M. VO_2 İnce Film İçeren İki Boyutlu ve Çok Katmanlı Yapılardaki Arayüzey Gerilmelerinin Termokromik ve Fotodetektör Performanslara Etkisi. Master's Thesis, University of Necmettin Erbakan, Konya, Turkey, August 2020.
6. Basyooni, M.A.M.A.; Shaban, M.; El Sayed, A.M.A.M. Enhanced Gas Sensing Properties of Spin-coated Na-doped ZnO Nanostructured Films. *Sci. Rep.* **2017**, *7*, 41716. https://doi.org/10.1038/srep41716.
7. Shportko, K.V. Disorder and compositional dependences in Urbach-Martienssen tails in amorphous $(GeTe)_x(Sb_2Te_3)_{1-x}$ alloys. *Sci. Rep.* **2019**, *9*, 6030. https://doi.org/10.1038/s41598-019-42634-8.
8. Bonalde, I.; Medina, E.; Rodríguez, M.; Wasim, S.M.; Marín, G.; Rincón, C.; Rincón, A.; Torres, C. Urbach tail, disorder, and localized modes in ternary semiconductors. *Phys. Rev. B* **2004**, *69*, 195201. https://doi.org/10.1103/PhysRevB.69.195201.
9. Mishra, V.; Sagdeo, A.; Warshi, K.; Rai, H.M.; Saxena, S.; Kumar, R.; Sagdeo, P. Metastable behavior of Urbach tail states in $BaTiO_3$ across phase transition. *arXiv* 2016, arXiv:1612.06756. Available online: https://arxiv.org/abs/1612.06756v1 (accessed on 1 March 2022).
10. Yoffe, A.D. Theory of Defects in Solids: Electronic Structure Defects in Insulators and Semiconductors. *Phys. Bull.* **1986**, *37*, 266–267. https://doi.org/10.1088/0031-9112/37/6/029.
